# Fast recovery from treatment resistant depression: clinical case from HIPOCRAT-ESK study, a cohort, non-interventional, prospective, real-world study of functional remission in TRD patients treated with Esketamine nasal spray

**DOI:** 10.1192/j.eurpsy.2026.11399

**Published:** 2026-07-31

**Authors:** T. Caraballo Lopez, M. P. Sierra San Miguel, Y. Cañada Pérez, P. Benavent Rodríguez, B. Nebot Tarazona

**Affiliations:** 1 Johnson & Johnson, Madrid; 2 Psychiatry, Hospital Universitario y Politécnico La Fe, Valencia, Spain

## Abstract

**Introduction:**

Treatment-resistant depression (TRD) represents a major challenge in psychiatry, particularly in patients with multiple recurrent episodes and poor functional outcomes. Esketamine nasal spray (ESK NS), a rapid-acting antidepressant targeting the glutamatergic system, has been approved as an add-on therapy for TRD. ESK NS has demonstrate both rapid symptom relief and functional improvement in real world and clinical trials.

**Objectives:**

We present the case of a 62-year-old woman with recurrent resistant major depressive disorder (MDD), currently participating in the HIPOCRATES-ESK study, who achieved ultra-rapid clinical and functional recovery during the first month of induction treatment with ESK NS.

**Methods:**

HIPOCRAT-ESK is a cohort, non-interventional, prospective, real-world study that evaluates functional remission after ESK NS initiation as primary endpoint. Rate of recovered from depression patients is included as secondary endpoint. Patients achieving both clinical remission (Montgomery-Åsberg Depression Rating Scale [MADRS] total score ≤ 10), and functional remission (Sheehan Disability Scale [SDS] total score ≤ 6) in a study visit are considered as recovered.

**Results:**

Our patient was first diagnosed with MDD in 2002 and, by 2025, had already experienced 20 major depressive episodes. At study baseline, she presented with severe depressive symptoms (mean MADRS total score of 37), and significant functional disability (mean SDS total score of 30). She initiated treatment with intranasal esketamine in combination with duloxetine, escitalopram, mirtazapine, and clorazepate dipotassium. Safety monitoring included assessment of vital signs, dissociative symptoms, and suicidality. After completing the one-month esketamine induction phase, the patient showed a dramatic reduction in depressive symptoms (mean MADRS total score of 8), achieving, therefore, clinical remission. Functional outcomes improved in parallel, mean SDS total score of 2 at the end of the induction phase (consistent with functional remission). The treatment was well tolerated (patient showed dissociative symptoms during the third administration that were managed with ESK NS dose decrease from 84 mg to 56 mg), and no adverse events required discontinuation.

**Conclusions:**

This is the first documented case of ultra-rapid recovery from recurrent MDD reported within the HIPOCRATES-ESK study, the first real-world prospective study in Spain. The case illustrates the potential of esketamine nasal spray, when used in combination with standard pharmacotherapy, to achieve comprehensive recovery in TRD. These findings support the role of ESK NS, not only in symptom reduction, but also in restoring functional outcomes, reinforcing its relevance as a transformative treatment option.

**Disclosure of Interest:**

T. Caraballo Lopez Employee of: Works as full-time employee in Johnson & Johnson, M. P. Sierra San Miguel Grant / Research support from: Has recieved educational and research grants from Johnson & Johnson, Consultant of: Has worked as scientific consultant for Johnson & Johnson, Y. Cañada Pérez Grant / Research support from: Has recieved educational and research grants from Johnson & Johnson, P. Benavent Rodríguez Grant / Research support from: Has recieved educational and research grants from Johnson & Johnson, B. Nebot Tarazona Employee of: Works as full-time employee of Johnson & Johnson

